# Plasma parasitemia as assessed by quantitative PCR in relation to clinical disease severity in African adults with falciparum malaria with and without HIV co-infection

**DOI:** 10.1007/s15010-020-01399-3

**Published:** 2020-02-19

**Authors:** Aase Berg, Sam Patel, Marit G. Tellevik, Christel G. Haanshuus, Ingvild Dalen, Kari Otterdal, Thor Ueland, Sabrina J. Moyo, Pål Aukrust, Nina Langeland

**Affiliations:** 1 Department of Medicine, Stavanger University Hospital, PO Box 8100, 4068 Stavanger, Norway; 2 Department of Medicine, Central Hospital of Maputo, Maputo, Mozambique; 3 Norwegian National Advisory Unit On Tropical Infectious Diseases, Department of Medicine, Haukeland University Hospital, 5021 Bergen, Norway; 4 Department of Research, Stavanger University Hospital, 4011 Stavanger, Norway; 5 Research Institute of Internal Medicine, Oslo University Hospital, Rikshospitalet, 0372 Oslo, Norway; 6 K. G. Jepsen Inflammatory Research Centre, University of Oslo, 0424 Oslo, Norway; 7 Faculty of Medicine, University of Oslo, 0316 Oslo, Norway; 8 Faculty of Health Sciences, University of Tromsø, 9037 Tromsø, Norway; 9 Department of Clinical Science, University of Bergen, 5021 Bergen, Norway; 10 Department of Microbiology and Immunology, Muhimbili University of Health and Allied Sciences, Dar es Salaam, Tanzania; 11 Section of Clinical Immunology and Infectious Diseases, Oslo University Hospital Rikshospitalet, 0372 Oslo, Norway; 12 Haraldsplass Deaconess Hospital, Bergen, Norway

**Keywords:** Falciparum malaria, Severe malaria, Quantitative plasma PCR, qPCR, Malaria disease severity, HIV co-infection, Mozambique

## Abstract

**Purpose:**

When considering malaria disease severity, estimation of parasitemia in erythrocytes is important, but sometimes misleading, since the infected erythrocytes may be sequestered in peripheral capillaries. In African children and Asian adults with falciparum malaria, parasitemia as assessed by quantitative PCR (qPCR) in plasma seems to be a valuable indicator of disease severity, but data on African adults as well as the impact of co-infection with HIV is lacking.

**Methods:**

In 131 patients with falciparum malaria in a public tertiary teaching hospital in Mozambique, plasma malaria parasitemia as assessed by qPCR, compared to qualitative malaria PCR in blood cell fraction, was related to malaria disease severity and HIV co-infection.

**Results:**

Of the 131 patients with falciparum malaria, based on positive qualitative PCR in the blood cell fraction, 93 patients (72%) had positive malaria qPCR in plasma. Patients with severe malaria as defined by the WHO criteria had higher malaria quantitative plasma parasitemia (median 143 genomes/µL) compared to those with uncomplicated malaria (median 55 genomes/µL, *p* = 0.037) in univariate analysis, but this difference was attenuated after adjusting for age, sex and HIV co-infection (*p* = 0.055). A quarter of the patients with severe malaria had negative qPCR in plasma.

**Conclusions:**

This study of adult African in-patients with falciparum malaria with and without HIV co-infection, neither confirms nor rejects previous studies of malaria qPCR in plasma as an indicator of disease severity in patients with falciparum malaria. There is a need for further and larger studies to clarify if parasitemia as assessed malaria qPCR in plasma could be a surrogate marker of disease severity in falciparum malaria.

## Introduction

In spite of decreasing incidence, falciparum malaria still causes about half a million fatalities every year, whereof 93% occur in sub-Saharan Africa and about two thirds are children below 5 years of age [1]. When considering disease severity in malaria patients, estimation of the parasitemia in the erythrocytes is important, but sometimes misleading. Thus, a low-level parasitemia as assessed by blood smear evaluation may be (1) an indicator of uncomplicated malaria, (2) an asymptomatic malaria carrier suffering from another potential severe infection, particularly in patients with HIV (human immunodeficiency virus) co-infection, or of (3) severe malaria with peripheral sequestration of the infected erythrocytes [2, 3].

In African children in a moderate-to-high transmission area, histidine-rich-protein 2 (HRP 2)-concentration in plasma distinguished severe malaria from coincidental parasitemia due to carrier state [4]. Similar, a multi-centre study of Asian adults and African children found that plasma HRP2 was a better indicator of severe malaria compared to parasite densities by microscopy (thick and thin slides). The same applied when they compared plasma parasitemia as assessed by qPCR (quantitative malaria polymerase chain reaction) methods to the mentioned microscopy [5]. Currently there are several published DNA (deoxyribonucleic acid) based qPCR methods for quantitation of *P. falciparum* [6]. Most of these methods are highly sensitive for detection of malaria and can detect the parasite even at very low levels (i.e., 0.03 parasites/µL present in blood) [6, 7], reflecting that most of the methods target a multi-copy gene e.g. mitochondria 20–160 copies per genome [8], TARE-2 250–280 copies per genome [9]. This means that a sensitive PCR has the potential to detect low density of malaria DNA produced by premunition, early infections, dormant stages, gametocytes, persistent clones, and destroyed parasites [10]. Therefore, highly sensitive methods are best for the purpose of detection and elimination of malaria. On the other hand, when measuring malaria severity, a method with high specificity is more preferable, even if it should be less sensitive. The mentioned Imwong study used a qPCR method targeting 18S ribosomal RNA. This gene target exists in five to eight copies per genome [6]. However, they examined African children and Asian adults and they did not consider HIV co-infection, which is an important factor in relation to disease severity in falciparum malaria [11–13]. We have previously reported outcome data and markers of inflammation in a cohort of adult patients with falciparum malaria with and without co-infection with HIV from Mozambique [12, 14]. Herein, we analysed the relationship between quantitative parasite DNA in plasma as a surrogate marker of parasitemia and clinical disease severity in patients with falciparum malaria with and without co-infection with HIV.

## Materials and methods

### Study design and participants

The study design has previously been described [12]. Briefly, clinical data and blood samples were collected prospectively on admission from all (*n* = 212) patients consecutively admitted during regular work hours during weekdays during two malaria seasons from January to March 2011 and from November 2011 to March 2012 in the Central Hospital of Maputo, Mozambique [12]. Inclusion criteria were age ≥ 18 years, negative pregnancy test in women in fertile age, axillary temperature ≥ 38 °C and/or clinical suspected malaria and written or fingerprinted informed consent from patient or if mentally confused or unconscious patient, from next of kin. “Clinical suspicion of malaria” was defined when the patient had a history of fever, chills, headache, mental confusion, dyspnoea, vomiting and/or diarrhoea, myalgia and/or general malaise in the absence of other symptoms and findings upon clinical examination or additional tests indicating other severe infections or conditions. Exclusion criteria were age < 18 years, pregnancy or mental confusion without relatives present to give consent. Baseline characteristics of the malaria patients are given in Table 1. Details on the study population, clinical data collection and additional examinations are described elsewhere [12].

**Table 1:** Clinical characteristics of adult inpatients with falciparum malaria (*n* = 131) with ( +) and without (–) HIV co-infection

Characteristics^a^	HIV + (*n* = 70)		HIV− (*n* = 61)	
	Uncomplicated (*n* = 13)	Severe (*n* = 57)	Uncomplicated (*n* = 33)	Severe (*n* = 28)
Age, years	38 (26–56)	40 (20–65)	39 (18–79)	41 (20–65)
Female, sex	6 (46%)	29 (51%)	13 (39%)	12 (43%)
Haemoglobin, g/dL	10.8 (7.7–13.8)	9.0 (2.5–15.7)	11.5 (5.6–15.7)	10.8 (3.2–17.0)
Platelets, × 10^9^/L	97 (23–205)	92 (8–330)	124 (21–324)	124 (11–452)
Se-Creatinine, µmol/L	101 (71–146)	257 (62–1529)	108 (57–203)	149 (72–357)
Malaria Rapid Test pos	12 (92%)	46 (81%)	32 (97%)	20 (71%)
Microscopy pos	10 (77%)	46 (81%)	28 (85%)	22 (79%)
Qual. blood cell mal. PCR pos	13 (100%)	56 (98%)^b^	32 (97%)	27 (96%)
Quant. plasma mal. PCR pos	8 (62%)	41 (72%)^2)^	22 (67%)	22 (79%)
Plasma *P.falciparum* DNA conc.^c^				
Mean genom./µL	82 (6–404)	1063 (6–17050)	125 (6–1210)	3965 (6–106500)
Median genom./µL^d^	26 (6–142)	48 (6–379)	11 (6–68)	43 (10–224)
Respiratory rate	21 (18–28)	26 (16–44)	20 (12–28)	25 (16–68)
BP systolic, mmHg	122 (100–170)	113 (80–170)	125 (90–240)	119 (70–160)
Bleeding disturbance/haemolysis	0	9	0	1
Died	0	9	0	1

^a^Values in mean (min–max) or number and percentage except

^b^One patient missing

^c^Which is in median and IQR interquartile range (25 and 75-percentiles)

^d^*n* = 93

### Procedures

The blood samples were collected from a pre-alcohol-cleaned peripheral vein into pyrogenic-free tubes with EDTA (Ethylene-diamine-tetra-acetic acid). The EDTA vacutainer tubes were turned gently, placed immediately on melting ice, and centrifuged within 30 min at 2000 rpm for 20 min. Plasma was aliquoted and stored first at − 20 °C for 24 h; then at − 80 °C until further analyses. The remaining blood cell fraction was also stored at − 80 °C. From this, we later purified the total nucleic acids using a MagNA Pure (Roche) robot and analysed with a qualitative PCR analysis for *Plasmodium*, HIV-1 and HIV-2.

According to the hospital’s routine and consistent with the standard procedures in the hospital’s laboratory, we performed malaria antigen test (HRP-2 Rapid Diagnostic Test (RDT)) for malaria in 2010–2011 using First Response® Malaria antigen *P. falciparum*, Premium Medical Corporation Ltd., Daman, India and in 2011–2012 ICT Malaria P.f.®, using ICT Diagnostics Cape Town, South Africa, thick smears (Giemsa 20% for 5 min) and HIV testing (Determine, Alere Medical Co. Ltd; Chiba, Japan and Unigold, Trinity Biotech plc, Bray, Ireland) and other routine blood tests (Haemoglobin, WBC, platelets, creatinine, bilirubin, AST, ALT, ALP and ESR).

Severe malaria was defined according to WHO (World Health Organization) criteria [15] and adjusted for what was possible to observe or measure in this clinical setting, i.e. one or more of the following nine severity criteria: Anemia with haemoglobin < 5 g/dL, observed bleeding disturbances or haemolysis, hypoglycaemia with glucose ≤ 2.2 mmol/L, renal failure with creatinine > 265 µmol/L, liver failure with observed jaundice or bilirubin > 50 µmol/L, signs of cerebral malaria with Glasgow Coma Scale < 11 and/or convulsions/confusion, hypotension with systolic blood pressure < 80 mmHg, signs of respiratory failure with respiratory rate > 30 and/or chest X-ray indicating respiratory insufficiency or severe prostration or hyperparasitemia > 10% as assessed by thick blood smears.

### PCR assessing *plasmodium* DNA extracted from the blood cell fraction

A conventional genus-specific malaria PCR, targeting the *Cytochrome b* gene on the mitochondrial genome, was performed on the extracted DNA from EDTA blood cell fractions. For positive samples the *Plasmodium* species was determined by conventional species-specific 18S PCR or divergent results by sequencing [14].

### Quantitative PCR assessing *P. falciparum* DNA directly from plasma

The concentration of *P. falciparum* DNA in plasma was measured by quantitative real-time PCR (qPCR) as described elsewhere [16] using Primer Pf-1 (5′-ATT GCT TTT GAG AGG TTT TGT TAC TTT-3′), primer Pf-2 (5′-GCT GTA GTA TTC AAA CAC AAT GAA CTC AA-3′) and probe Pf (5′-CAT AAC AGA CGG GTA GTC AT-3′) (Applied Biosystems, Cheshire, UK). Each PCR test was performed in a 10-µL reaction mixture and qPCR assay was performed using a LightCycler 480 Instrument II (Roche Diagnostics, Mannheim, Germany). All samples were run on LightCycler® 480 Multiwell Plate 384, white (Roche), and sealed with LightCycler® 480 Sealing Foil (Roche). Each run included a positive control and multiple no-template controls. DNA extracted from an external reference material *P. falciparum* (US 03 F Benin I), containing exclusively ring stage parasites in a concentration of 2000 p/μL, was used for dilution series, fivefold, to prepare standard curve for estimating efficiency of the PCR and for quantification. DNA quantity for samples with *P. falciparum* DNA less than the Limit of Quantification (LOQ) was set to be equal to or less than the LOQ (estimated to ≤ 6.4 parasites/µL). Samples with a *P. falciparum* DNA concentration higher than the most concentrated standard were diluted and qPCR repeated. The qPCR was also repeated for samples with weak positive or uncertain results. The efficiency (using the formula *E* = 10^–1/slope^ – 1) was 91.2%, and Error value (*E*) of the assay 0.012. A unidirectional workflow pre- to post-PCR was enforced, and preparation of PCR reaction mixture, DNA preparations and PCR were carried out in facilities physically separated from each other.

### Statistics

Descriptive statistics are presented as mean values, ranges (min–max), medians, interquartile ranges (IQR; 25–75 percentile), and as counts and percentages. Histograms and Box plots were used for illustration. Comparison of patients with severe malaria as assessed by WHO criteria with and without positive qPCR in plasma was done with Chi-squared tests for dichotomous variables and Mann Whitney test for continuous variables. Association between patients with severe malaria and plasma qPCR, age, sex and HIV co-infection was evaluated with binary logistic regression analysis with 95% confidence intervals (CI) and *p* values from Wald tests. For the regression analysis, malaria qPCR values were log_10_ transformed and the assumption of linearity of the effect of log qPCR was tested with flexible modelling (fractional polynomials) in Stata version 16.0, the rest of the statistical analyses were performed in IBM SPSS v. 24.

## Results

### Previous study: patient population

The patient population is described earlier [12]. In summary, 131 patients had *P. falciparum* malaria, defined as positive qualitative malaria PCR in whole blood (*n* = 129) or positive thick slide and rapid test when qualitative PCR was not performed (*n* = 2). Only two patients had co-infection with *P. vivax* and *P. malariae,* respectively. Of the 131 falciparum malaria patients, there were 10 fatalities (8%), whereof nine were HIV positive. Owing to the low number of fatalities, mortality was not evaluated in further analyses. Seventy patients (53%) were co-infected with HIV, and there was a higher proportion of severe malaria cases among these (81%) than among the HIV negative patients (46%) (*p* < 0.001) [12]. Only 20 of the HIV patients (29%) were aware of their HIV status before admission, of which 13 were on antiretroviral therapy, whereof nine had effective treatment defined as undetectable HIV-RNA in plasma.

### Present study: quantitative malaria PCR in plasma

Malaria qPCR in plasma was positive for 93 of the 131 patients (71%). Of the 131 patients with positive qualitative *P. falciparum* PCR in blood cells, there was no significant difference between the 93 patients with positive qPCR in plasma and the 38 patients without, in relation to HIV status (71 vs. 72%, *p* = 0.89) nor to malaria severity (74 vs. 67%, *p* = 0.37). All the ten fatalities had severe malaria and the eight of them that had qPCR done, were all positive. Clinical characteristics are shown in Table 1.

**Fig. 1:**
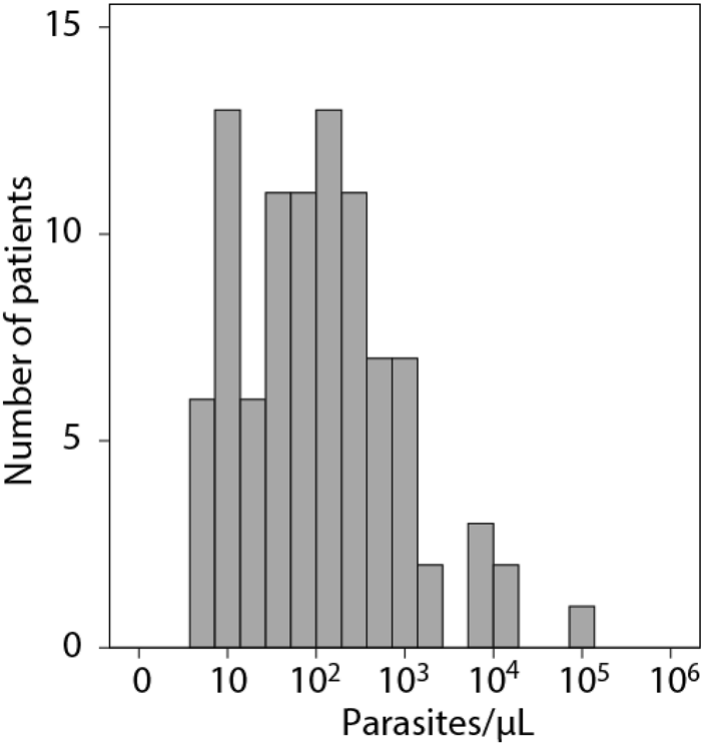
Distribution of plasma parasitemia as assessed by qPCR in patients with falciparum malaria on admission (*n* = 93, including deaths)

The quantitative plasma parasitemia distribution is seen in Fig. 1.

### Plasma parasitemia as assessed by qPCR in relation to disease severity and HIV co-infection

**Fig. 2:**
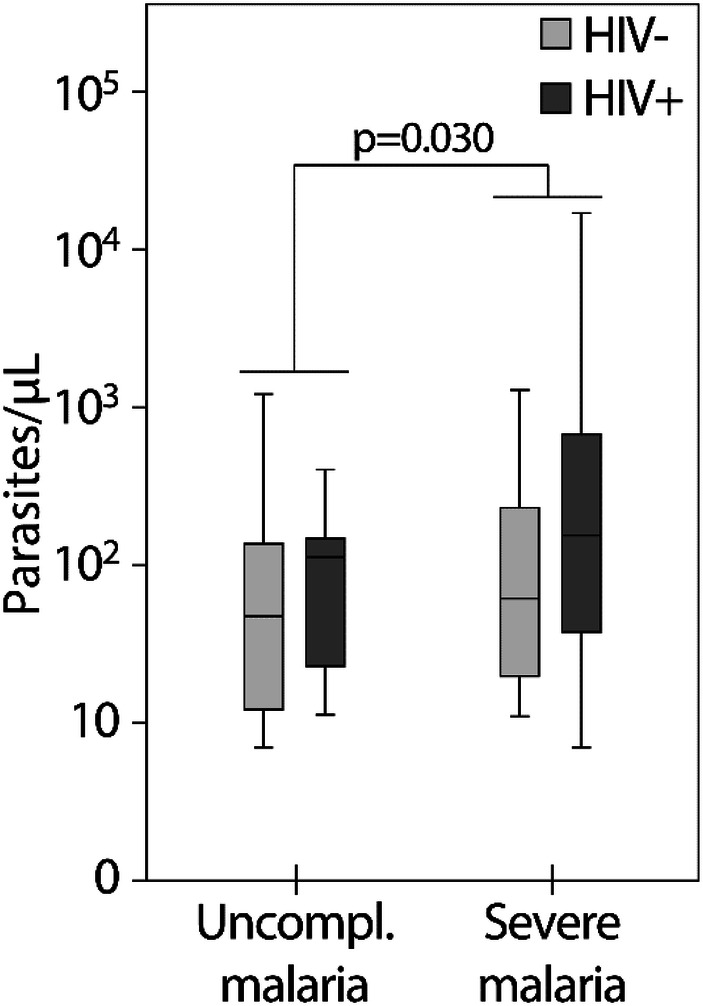
The qPCR related to severity and HIV co-infection (*n* = 93, including deaths)

There was a significant difference in the qPCR between patients with severe (*n* = 63) and uncomplicated malaria (*n* = 30) among the 93 patients with positive plasma qPCR (*p* = 0.030) (Fig. 2), with median (min–max) 143 (6–106,500) genomes/µL versus 55 (6–1210) genomes/µL. On the other hand, 26% (22/85) of the patients with severe malaria had negative quantitative plasma PCR, and those patients had significant more frequent severe anaemia compared to the ones with positive qPCR (*p* < 0.001). The severe malaria patients with positive plasma qPCR had significant more frequent severe liver failure and hyperparasitemia as assessed by thick blood smears, and more pronounced thrombocytopenia (Table 2).

**Table 2:** Comparison of severe malaria patients with and without qPCR (*n* = 85)

*n*	Plasma-PCR pos	Plasma-PCR neg	*p*^a^
	63	22	
Severe anaemia^b^	5 (3/59)	45 (10/22)	< 0.001
Platelets^c^	84 (8–319)	151 (17–452)	0.002
Severe renal failure^d^	24 (13/54)	32 (6/19)	0.52
Severe respiratory failure^e^	25 (16/63)	27 (6/22)	0.86
Hypoglycaemia^f^	9 (5/54)	0 (0/19)	0.17
Hypotension^g^	2 (1/54)	0 (0/21)	0.53
Coagulation disturbance^h^	8 (5/63)	23 (5/22)	0.064
Cerebral malaria^i^	37 (23/63)	27 (6/22)	0.43
Hyperparasitemia^j^	76 (45/59)	21 (4/19)	< 0.001
Liver failure^k^	25 (16/63)	0 (0/22)	0.009
Fatalities	13 (8/63)	10 (2/21)	0.70

The data are percentage (proportion of patients with given condition/the patients observed), except for the platelets given in mean (min–max)

^a^The p-values are from Chi-squared tests (dichotomous data) and Mann–Whitney test (platelets)

^b^Defined as Hb < 5 g/dL

^c^Platelets, × 10^9^/L

^d^Defined as Creatinine > 265µmol/L

^e^Defined as respiratory rate > 30/min or CXR with respiratory insufficiency

^f^Defined as Se-glucosis < 2.2mmol/L

^g^Systolic blood pressure < 80 mmHg

^h^Bleeding disturbance or hemolysis

^i^Glasgow Coma Scale < 11 and/or convulsions

^j^Microscopy of thick slide with parasitemia > 4 +

^k^Bilirubin > 50 µmol/L and/or jaundice

In binary logistic regression analyses the strongest predictor for severe malaria was HIV co-infection with *p* < 0.001 and an OR of 5.36, indicating that a HIV co-infected malaria patient is about five times more likely to develop severe malaria than a HIV negative patient, adjusting for age, sex and qPCR (Table 3). Plasma qPCR as surrogate marker for plasma parasitemia was associated with severe malaria (*p* = 0.037), but this association was attenuated when adjusting for age, sex and HIV co-infection (*p* = 0.055), even if the OR was about the same before and after these adjustments (OR 1.41 and 1.43, respectively). The difference in plasma qPCR between HIV positive (*n* = 49) and HIV negative patients (*n* = 44) was not significant (143 [37–593] genomes/µL vs. 55 [12–224] genomes/µL, HIV-seropositive and HIV-seronegative, respectively, *p* = 0.068).

**Table 3:** Association between malaria severity and qPCR, age, sex and HIV co-infection in 131 malaria patients

Characteristics	Unadjusted		Adjusted	
	OR (95% CI)	*p* value^a^	OR (95% CI)	*p* value
Age	1.01 (0.99, 1.04)	0.40	1.01 (0.98, 1.04)	0.66
Sex	0.76 (0.37, 1.56)	0.45	0.72 (0.31, 1.67)	0.45
HIV	5.17 (2.36, 11.33)	< 0.001	5.37 (2.37, 12.16)	< 0.001
Log qPCR	1.41 (1.02, 1.96)	0.037	1.43 (0.99, 2.07)	0.055

Results from binary logistic regression analysis

*OR* odds ratio, *CI*  confidence interval

^a^The *p* values are from Wald tests

No significant differences according to disease severity was found in the 131 malaria patients by the use of positive thick blood smears (*p* = 0.97) or malaria antigen test (*p* = 0.062).

## Discussion

Previously, a sub-study of the AQUAMAT multicentre study of artesunate versus quinine treatment for severe malaria [5] reported an association between disease severity and *P. falciparum* levels as assessed by qPCR in plasma in adult patients from Bangladesh and India and in children from Tanzania and Mozambique. The present study is, however, the first report on qPCR assessment in patients with falciparum malaria in African adults, and importantly, the present study also included patients co-infected with HIV. Similar to the Aquamat sub-study there was a significant association between disease severity and qPCR not observed for positive thick blood smears and malaria antigen test. However, whereas the Aquamat sub-study did not consider HIV co-infection, we found that the association between plasma parasitemia as assessed by qPCR and severe malaria was attenuated and not significant after adjusting for age, sex and HIV co-infection, even if the OR remained about the same. This is probably due to the relatively low number of patients in each subgroup. An alternative explanation could be that HIV co-infection increases the malaria disease severity through other mechanism not directly related to increasing the malaria qPCR in plasma. This is less plausible since other investigators found increased malaria parasitemia as least in whole blood as assessed by different methods including blood smears in relation to HIV infection [11, 17, 18]. Second, the observed increased severity may be due to other concomitant HIV-associated opportunistic co-infections difficult to diagnose in this relative low resource setting [3, 19]. This may have been the case, considering the substantial overlap of HIV and malaria prevalence in this region. On the other hand, with increasing coverage of antiretroviral therapy (ART) in sub-Saharan Africa, the mentioned bias may be diminished by examining this interaction in a larger group of asymptomatic HIV infected patients with fully suppressed viral load on anti-retroviral therapy.

Twenty-six percent of the patients with severe malaria (22 of 85) had parasitemia below the limit of detection for the quantitative plasma PCR. As the parasite has erythrocytes as its major target cells in whole blood, it is not surprising that PCR analyses from whole blood are in general more sensitive than PCR analyses from plasma. A former analysis by RT-PCR for the 18S ribosomal gene for falciparum malaria in small children also found 3.5-fold higher parasitemia in whole blood compared to plasma [20]. The malaria PCR from whole blood is more sensitive in diagnosing low-level parasitemia, as for instance in vaccine research, but harbours the risk of over-diagnosing disease in asymptomatic carriers [21]. This explains the pronounced difference in plasma and red cell parasitemia, but does not explain the 22 patients diagnosed as severe malaria without having positive plasma qPCR. The severe malaria patients with plasma qPCR had significant more frequent severe liver failure and hyperparasitemia as assessed by thick blood smears, and also thrombocytopenia, but no significant difference in the other criteria for severe malaria.

This study cannot confirm nor oppose the results of the Aquamat sub-study. On the other hand, this study underlines the importance of considering HIV co-infection in malaria. With the great geographic overlap of HIV and malaria together with the increased risk of severe malaria, HIV status should always be included. There is need for further studies to clarify if qPCR may be a useful method for quantification of parasite load in patients with falciparum malaria as an indication of very severe malaria. Provided that the sensitivity of method is improved, it is possible that qPCR will have a future potential for evaluating disease severity in falciparum malaria, and there is a need for further and larger studies to clarify whether quantitative qPCR may be an indicator of clinical disease severity in falciparum malaria.

## Abbreviations

PCR: Polymerase chain reaction
qPCR: Quantitative PCR
HIV: Human immunodeficiency virus
HRP 2: Histidine rich protein 2
DNA: Deoxyribonucleic acid
TARE 2: Telomere-associated repetitive element 2
RNA: Ribonucleic acid
EDTA: Ethylene-diamine-tetra-acetic acid
RDT: Rapid Diagnostic Test for malaria
WBC: White blood cells
AST: Aspartate transaminase
ALT: Alanine transaminase
ALP: Alkaline phosphatase
ESR: Erythrocyte sedimentation rate
WHO: World Health Organization
LOQ: Limit of quantification
IQR: Interquartile ranges
CI: Confidence intervals
AQUAMAT: Artesunat versus quinine in the treatment of severe falciparum malaria in African children
ART: Antiretroviral therapy
